# Socioeconomic and Racial/Ethnic Disparities in Cancer Mortality, Incidence, and Survival in the United States, 1950–2014: Over Six Decades of Changing Patterns and Widening Inequalities

**DOI:** 10.1155/2017/2819372

**Published:** 2017-03-20

**Authors:** Gopal K. Singh, Ahmedin Jemal

**Affiliations:** ^1^US Department of Health and Human Services, Office of Health Equity, Health Resources and Services Administration, 5600 Fishers Lane, Room 13N42, Rockville, MD 20857, USA; ^2^American Cancer Society, Inc., Surveillance & Health Services Research, 250 Williams Street NW, Corporate Center, Atlanta, GA 30303, USA

## Abstract

We analyzed socioeconomic and racial/ethnic disparities in US mortality, incidence, and survival rates from all-cancers combined and major cancers from 1950 to 2014. Census-based deprivation indices were linked to national mortality and cancer data for area-based socioeconomic patterns in mortality, incidence, and survival. The National Longitudinal Mortality Study was used to analyze individual-level socioeconomic and racial/ethnic patterns in mortality. Rates, risk-ratios, least squares, log-linear, and Cox regression were used to examine trends and differentials. Socioeconomic patterns in all-cancer, lung, and colorectal cancer mortality changed dramatically over time. Individuals in more deprived areas or lower education and income groups had higher mortality and incidence rates than their more affluent counterparts, with excess risk being particularly marked for lung, colorectal, cervical, stomach, and liver cancer. Education and income inequalities in mortality from all-cancers, lung, prostate, and cervical cancer increased during 1979–2011. Socioeconomic inequalities in cancer mortality widened as mortality in lower socioeconomic groups/areas declined more slowly. Mortality was higher among Blacks and lower among Asian/Pacific Islanders and Hispanics than Whites. Cancer patient survival was significantly lower in more deprived neighborhoods and among most ethnic-minority groups. Cancer mortality and incidence disparities may reflect inequalities in smoking, obesity, physical inactivity, diet, alcohol use, screening, and treatment.

## 1. Introduction

Monitoring and reducing health disparities according to socioeconomic status (SES) and race/ethnicity have long been an important health policy goal in the United States [1–3]. Studies have shown the dynamic nature of socioeconomic disparities in cancer rates as the association between SES and incidence and mortality from major cancers has changed markedly during the past 5 decades [4–7]. Temporal patterns have changed largely as a result of differential rates of decline or increase in mortality among those in various socioeconomic groups and changing sociodemographic patterns in major cancer risk factors such as smoking, diet, obesity, and physical inactivity [3–6].

Association between cancer mortality/incidence and SES varies by cancer type [3–16]. Contemporary data indicate lower rates of lung, stomach, liver, cervical, esophageal, and oropharyngeal cancer and higher rates of breast cancer and melanoma at higher SES levels [3–18]. The major behavioral determinants of cancer, such as smoking, diet, alcohol use, obesity, physical inactivity, reproductive behavior, occupational and environmental exposures, and cancer screening, are themselves substantially influenced by individual-level and area-level socioeconomic factors [2, 3, 6, 7, 15, 18–20].

Analyzing socioeconomic and racial/ethnic patterns in cancer mortality and incidence is important because it allows us to quantify cancer-related health disparities between the least- and most-advantaged social groups and to identify areas or population groups that are at greatest risk of cancer diagnosis and mortality and who may therefore benefit from targeted social and medical interventions [3, 6]. Temporal analyses can be used to track progress toward reducing health disparities as recent cancer disparities can be compared with those that existed in the previous decades [3, 6]. Comparison of cancer trends across population groups or areas may provide important insights into the impact of cancer control interventions, such as smoking cessation, cancer screening, physical activity campaigns, and cancer treatment [3–7, 10].

Reliable individual-level SES data for all ages, especially for ages 65 and older, are lacking on US death certificates, which provide the basis for computing cancer mortality rates for various demographic groups and geographic areas [3–7, 10, 21]. Individual-level data on education and income are not available for cancer patients in the Surveillance, Epidemiology, and End Results (SEER) database, which has been the primary source of data on cancer incidence, stage at diagnosis, treatment, and survival patterns in the US for the past 4 decades [6, 10, 16, 22]. Because of these data limitations, population-based studies of socioeconomic disparities in cancer rates have utilized area-based socioeconomic data linked to both individual- and aggregate-level cancer data [3–7, 10, 14, 17, 18]. Recent linkages of the Census and Current Population Survey (CPS) records with the National Death Index and cancer patient medical records have led to the development of longitudinal, cohort databases, allowing estimation of cancer incidence, mortality, disease stage, and survival patterns according to individual-level socioeconomic characteristics [11, 16, 23, 24].

In this paper, we extend previous analyses by examining the extent to which long-term cancer disparities in the US have changed during the last six decades. We use a census-based deprivation index to examine temporal area socioeconomic and racial/ethnic disparities in US all-cancer, lung, colorectal, prostate, breast, and cervical cancer mortality and assess racial/ethnic and area-based socioeconomic patterns in cancer incidence and survival using the SEER database. Using prospectively linked census and mortality records, we analyze temporal individual-level racial/ethnic and socioeconomic inequalities in mortality from all-cancers combined and lung, colorectal, prostate, breast, cervical, stomach, liver, and esophageal cancers. Lung cancer is the leading cause of cancer mortality, and colorectal, prostate, and breast cancers are among the most commonly diagnosed cancers; these sites, along with stomach, liver, esophageal, and cervical cancer, contribute greatly to the overall cancer burden in the US [3, 21, 22, 25, 26]. Taken together, these cancers account for more than half of all-cancer deaths and new cancer cases in the US [22, 25, 26].

## 2. Methods

We examined disparities in cancer mortality, incidence, and survival using three national data sources: the national mortality database, the 1979–2011 National Longitudinal Mortality Study (NLMS), and the SEER cancer registry database [2, 3, 11, 21–23]. The national mortality database has been the primary source of mortality analyses and surveillance by age, sex, race/ethnicity, cause of death, and place of residence for over a century [2, 3, 6, 21]. Since the vital-statistics-based national mortality database lacks reliable socioeconomic data for all ages, socioeconomic patterns in mortality were derived by linking county-level socioeconomic data from the 1970–2000 decennial censuses with the national mortality statistics [3–7, 10, 17]. The NLMS is a longitudinal dataset for examining socioeconomic, occupational, and demographic factors associated with all-cause and cause-specific mortality in the US [23]. The NLMS is conducted by the National Heart, Lung, and Blood Institute in collaboration with the Census Bureau, the National Cancer Institute (NCI), the National Institute on Aging, and the National Center for Health Statistics. The NLMS consists of 39 CPS and census cohorts between 1973 and 2011 whose survival (mortality) experiences were studied from 1979 to 2011 [23]. The NCI's SEER database included incidence and patient survival data from 11 population-based SEER cancer registries, including the states of Connecticut, Hawaii, Iowa, New Mexico, and Utah, and metropolitan areas of Atlanta, Detroit, Los Angeles, San Francisco-Oakland, San Jose-Monterey, and Seattle [22].

We used previously developed factor-based deprivation indices from the 1970, 1990, and 2000 decennial censuses and the 2008–2012 American Community Survey to examine temporal SES disparities in cancer mortality, incidence, and survival [3–5, 27, 28]. The county deprivation index consisted of 11 census-based social indicators, viewed as broadly representing educational opportunities, labor force skills, economic and housing conditions, and general living standards in a given county [3–5, 27]. Selected indicators of education, occupation, wealth, income distribution, unemployment rate, poverty rate, and housing quality were used to construct the index [3–5, 27]. The neighborhood (census tract) deprivation index consisted of 17 social indicators and was used in the incidence and survival analyses. Higher index scores denote higher levels of SES and lower levels of deprivation. Details of the US deprivation indices are provided elsewhere [3–5, 27].

Index scores were categorized into 5 area groups, ranging from being the most-deprived (first quintile) to the least-disadvantaged (fifth quintile) county or neighborhood groups [3–6, 27]. The 1970 SES/deprivation index was used to calculate mortality rates from 1950 to 1974; the 1990 deprivation index was used to calculate mortality rates from 1975 to 1998; the 2000 deprivation index was used to calculate mortality rates from 1999 to 2008; and the 2008–2012 index was used to calculate mortality rates from 2009 to 2014 [3–5]. For census tract-level socioeconomic patterns in incidence and survival, the 1990 deprivation index was linked to the 1988–1999 incidence data from 11 SEER cancer registries [6].

Cancer mortality and incidence rates for each county, area deprivation, or individual-level socioeconomic and racial/ethnic group were age-adjusted by the direct method using the 2000 US standard population [6, 17, 21, 22]. Weighted least squares regression models were fitted to county-level age-adjusted cancer mortality rates annually to estimate correlations of socioeconomic deprivation with all-cancer mortality from 1950 to 2014. Log-linear regression models were used to estimate annual rates of change in SES-specific mortality trends during 1950–2013 [17, 18]. Socioeconomic disparities in mortality and incidence were described by rate ratios (relative risks) and rate differences (absolute inequalities), which were tested for statistical significance at the 0.05 level. Trend tests across SES categories were conducted using both 0.01 and 0.05 levels of significance. Unless otherwise noted, the word “significant” denotes statistically significant difference at the 0.05 level. Cause-specific survival rates were computed for men and women diagnosed with malignant cancer during 1988–1999 who were followed for vital status through December 31, 1999 [22]. Disparities in SEER-based patient survival were analyzed by multivariate Cox regression models. Cohort-based mortality rates were derived from the NLMS data by using the person-years approach [16, 23].

## 3. Results

### 3.1. Area Socioeconomic and Racial Disparities in Cancer Mortality

**Figure 1:**
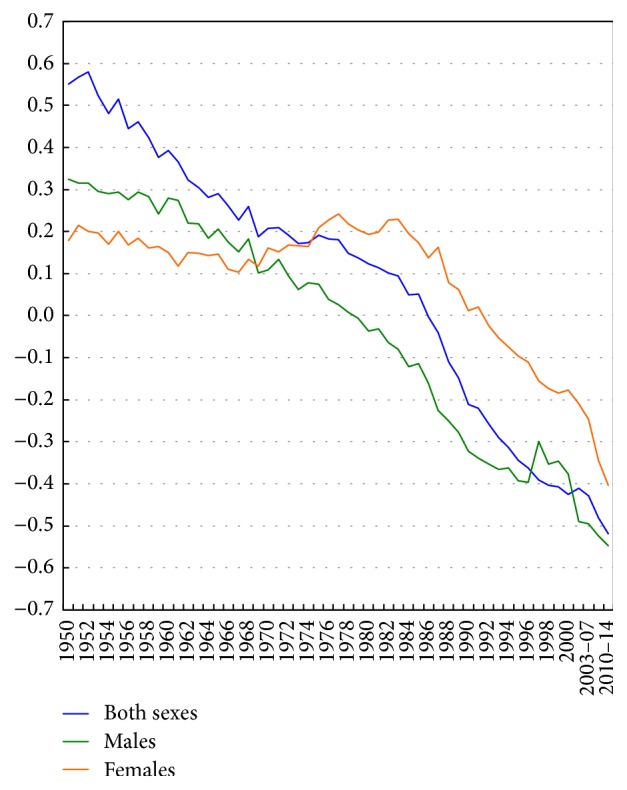
Weighted correlations between area socioeconomic index and county-level age-adjusted cancer mortality rates, United States, 1950–2014.

**Figure 2:**
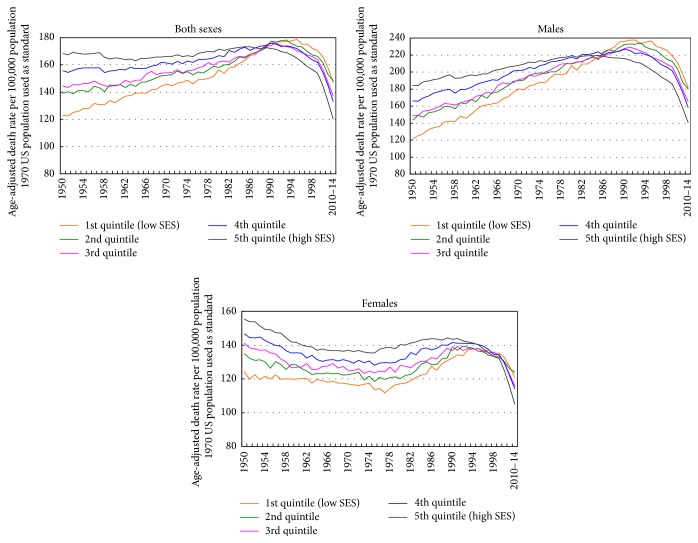
Trends in all-cancer mortality by area socioeconomic deprivation index, United States, 1950–2014.

Figures 1 and 2 show changing socioeconomic patterns in US all-cancer mortality rates over time. The correlation between area-level SES and all-cancer mortality rates changed from +0.55 in 1950 to −0.52 in 2014. The relationship between SES and all-cancer mortality rates reversed earlier for males than females. Between 1950 and 2014, the correlation changed from +0.33 to −0.55 for males and from +0.18 to −0.40 for females (Figure 1). Changing socioeconomic patterns in rates are more easily discernible in Figure 2, which shows a positive SES gradient in all-cancer mortality rates from 1950 through the mid-1980s and an increasingly inverse SES gradient since the mid-1990s. In 1950, those in the most-deprived group had 27% lower cancer mortality, but by 2010–2014, they had a 22% higher cancer mortality rate compared to those in the most-affluent group. Socioeconomic gradients and absolute inequalities were steeper for men than for women. In 2010–2014, compared to their counterparts in the least-deprived group, men had 29% higher cancer mortality and women 15% higher mortality in the most-deprived group (Figure 2).

**Figure 3:**
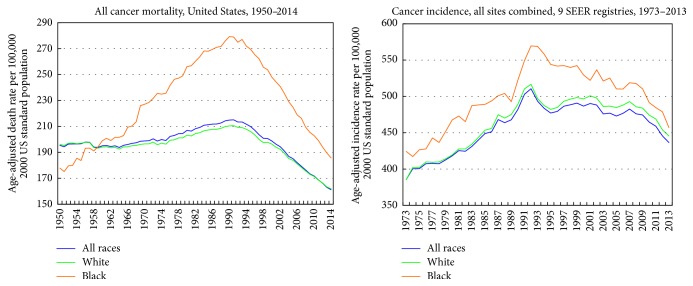
Trends in all-cancer mortality and incidence rates by race, United States, 1950–2014. The 9 SEER registries include San Francisco-Oakland, Connecticut, Detroit-Metropolitan, Hawaii, Iowa, New Mexico, Seattle-Puget Sound, Utah, and Atlanta-Metropolitan.

Long-term trends show a reversal of Black-White disparities in all-cancer mortality during 1950–2014 (Figure 3). During the early 1950s, Blacks/African Americans had lower all-cancer mortality than Whites. Since the 1960s, cancer mortality rates have been significantly higher for Blacks than for Whites. Cancer mortality rates for Blacks increased dramatically during 1950–1990. Since the early 1990s, mortality rates have declined for both Blacks and Whites (Figure 3).

**Figure 4:**
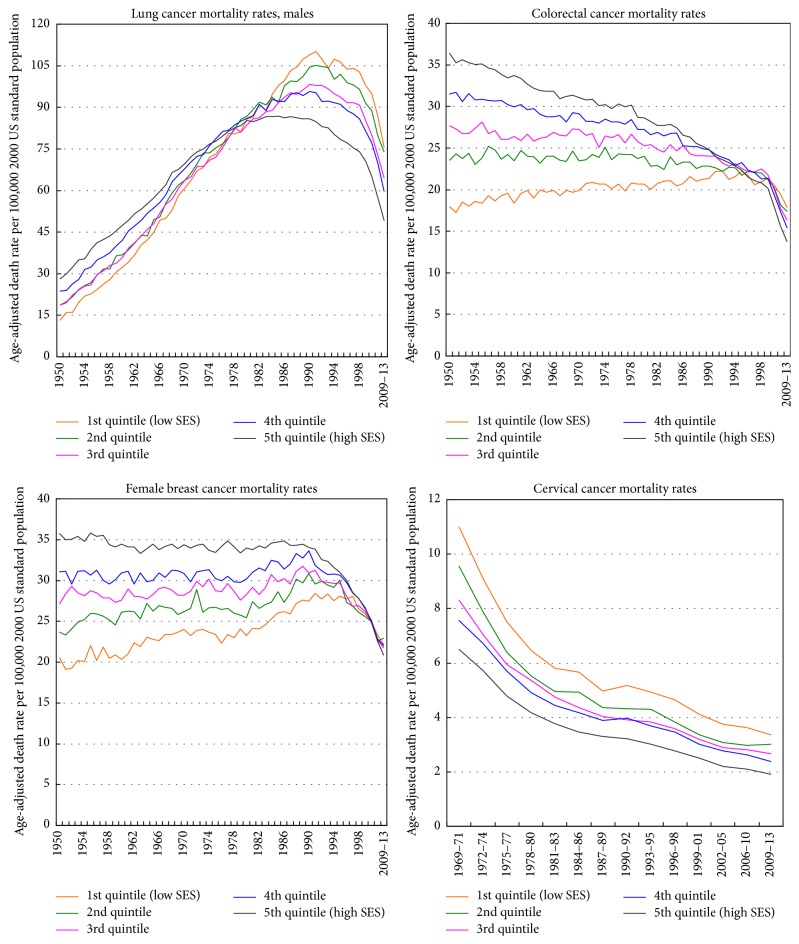
Trends in lung, colorectal, breast, and cervical cancer mortality rates by area socioeconomic deprivation index, United States, 1950–2013.

Socioeconomic trends in lung cancer mortality differed for men and women. From 1950 to 1974, men in more affluent areas had higher lung cancer mortality than those in more deprived areas. Socioeconomic differentials reversed and started to widen by the early 1980s for men and by 2002 for women (Figure 4). In 2009–2013, socioeconomic inequalities in lung cancer mortality were larger and more consistent for men than for women. Men and women in the most-deprived group had 54% and 16% higher lung cancer mortality rates than their most-affluent counterparts, respectively.

During 1950–1990, lung cancer mortality among men increased at 5.1% per year in the most-deprived group, significantly faster than the annual rate of increase of 2.8% for men in the most-affluent group. Moreover, during 1991–2013, lung cancer mortality fell at a more rapid pace for men in the more affluent groups (2.53% annually in the most-affluent group versus 1.61% in the most-deprived group). During 1950–2013, there were marked increases in lung cancer mortality among women in all deprivation groups, although the annual rate of increase in mortality was somewhat higher in the more deprived groups.

Socioeconomic trends in US colorectal cancer mortality changed dramatically between 1950 and 2013, with the positive SES gradients in mortality narrowing over time and then reversing at the turn of the 21st century (Figure 4). In 2009–2013, there was an inverse SES gradient, with those in the most-deprived group having a 30% higher colorectal cancer mortality rate than their most-affluent counterparts. During 1950–2013, colorectal cancer mortality increased at 0.25% per year in the most-deprived group, whereas it fell consistently in the higher SES groups; the annual rates of decline in mortality in the two most-affluent groups were 1.24% and 0.87%, respectively. Socioeconomic trends in colorectal cancer mortality were generally similar for men and women.

Prostate cancer mortality did not vary appreciably over time by area deprivation. However, during the past decade, an inverse socioeconomic gradient in prostate cancer mortality was found, with mortality rates falling similarly in all deprivation groups between 1995 and 2013. In 2009–2013, men in the most-deprived group had 19% higher prostate cancer mortality than men in the most-affluent group (data not shown).

Socioeconomic differences in breast cancer mortality narrowed over time and reversed during the past decade, as higher deprivation levels are now associated with higher breast cancer mortality rates. The reversal of the trend has occurred as breast cancer mortality rates have declined over time for more affluent women and have increased or remained stable for women in the more deprived groups. During 1950–2013, the breast cancer mortality rate increased by 0.54% annually for women in the most-deprived group, while it decreased by 0.48% per year for women in the most-affluent group. In 2009–2013, women in the most-deprived group had 6% higher mortality than their most-affluent counterparts. In 1950, women in the most-deprived group had 42% lower mortality than women in the most-affluent group (Figure 4).

Cervical cancer mortality rates in the US have declined consistently for the past 6 decades, and rates of mortality decline among women in all deprivation groups have been similar. However, despite the decline, substantial inverse socioeconomic gradients in cervical cancer mortality have persisted. In 2009–2013, women in the most-deprived group had a 76% higher cervical cancer mortality rate than their most-advantaged counterparts, a pattern of inequality that also characterized the trends during 1969–2008 (Figure 4).

### 3.2. Individual-Level Socioeconomic and Racial/Ethnic Disparities in Cancer Mortality

All-cancer mortality rates among men varied consistently by individual-level education and income levels, with gradients in mortality being more pronounced in 2003–2011 than during 1979–1998 (Tables 1 and 2). During 2003–2011, men with less than a high school education had 68% higher cancer mortality than those with a college degree, whereas men below the poverty level had 80% higher cancer mortality than men with incomes ≥600% of the poverty level (Table 2). Although higher cancer mortality was associated with lower education and income levels in women, the gradients were less marked in women than in men. All-cancer mortality rates were significantly higher among Blacks and lower among Asian/Pacific Islanders (APIs) and Hispanics compared to non-Hispanic Whites.

Socioeconomic inequalities in lung cancer mortality, especially among men, were quite marked. During 2003–2011, men with less than a high school education and those below the poverty level had 2.6 times higher lung cancer mortality than their more educated and affluent counterparts. Education and income levels were also inversely related to female lung cancer mortality. Education and income inequalities in lung cancer mortality increased over time. Lung cancer mortality rates were significantly lower among APIs and Hispanics but significantly higher among Black and American Indian/Alaska Native (AIAN) men, compared to non-Hispanic Whites.

Both education and income were significantly associated with colorectal cancer mortality; men and women with less than a high school education had, respectively, 42% and 120% higher mortality risks than those with a college degree. During 2003–2011, breast cancer mortality did not vary by education and income levels. During 2003–3011, men with low education and income were at increased risk of prostate cancer mortality (Table 2). Black men had more than the risk of prostate cancer mortality than non-Hispanic White men. Prostate and breast cancer mortality was markedly lower among APIs and Hispanics compared to non-Hispanic Whites.

There were steep education and income gradients in cervical cancer mortality. During 2003–2011, women with less than a high school education and below the poverty level had 6.3 and 4.0 times higher cervical cancer mortality than women with the highest education and income levels, respectively (Table 2). Rates of stomach, liver, and esophageal cancer mortality also varied substantially and inversely by education and income levels (Table 1). Stomach and liver cancer mortality rates were much higher among APIs, Hispanics, and Blacks compared to non-Hispanic Whites.

### 3.3. Disparities in Site-Specific Cancer Incidence

Consistent with the mortality trends, all-cancer incidence rates showed an upward trend among both Black and White Americans until the early 1990s and have been declining since then (Figure 3). As was the case with the mortality trends, Black Americans experienced higher rates of cancer incidence than White Americans during 1973–2013.

Socioeconomic patterns in cancer incidence are generally similar to those in cancer mortality [6, 9]. According to the 1988–1992 SEER data, higher neighborhood SES was associated with higher cancer incidence rates for the total population and for women in particular (Table 3). The male lung cancer incidence rate was 61% higher in the most-deprived than the least-deprived neighborhoods. The inverse SES gradient in male lung cancer incidence was observed for both White and Black men. Prostate cancer incidence rates increased with increasing neighborhood SES for both White and Black men. Men in the most-affluent neighborhoods had a 36% higher prostate cancer incidence rate than men in the most-deprived neighborhoods. Higher neighborhood SES levels were associated with higher breast cancer incidence rates in both White and Black women. Women in the most-affluent neighborhoods had 47% higher breast cancer incidence rates than their most-disadvantaged counterparts. Cervical cancer incidence increased consistently with increasing deprivation levels. Women in the most-deprived neighborhoods had a 2.7 higher risk of cervical cancer than women in the most-affluent neighborhoods. Higher deprivation levels were associated with higher rates of stomach, liver, and esophageal cancer incidence.

### 3.4. Disparities in Site-Specific Cancer Survival

**Figure 5:**
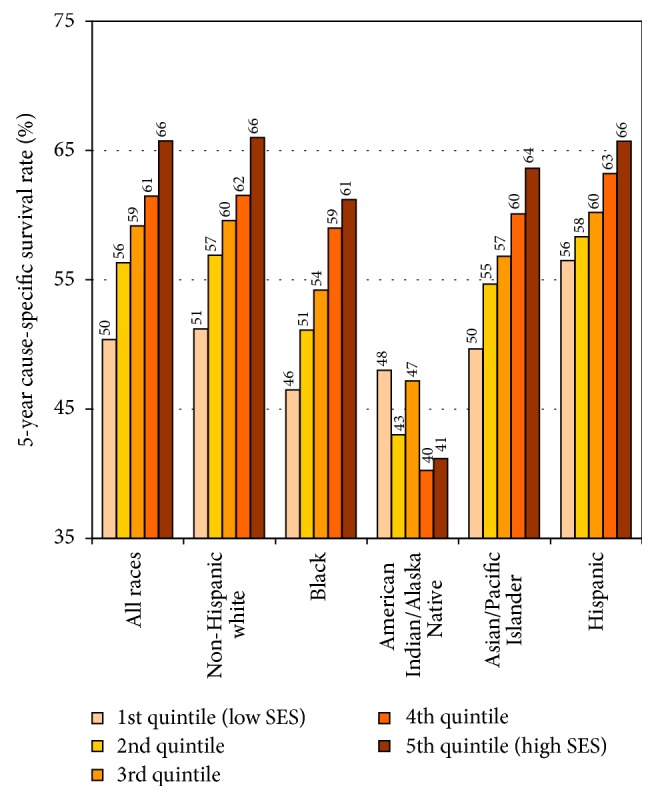
Cancer survival by census tract socioeconomic index and race/ethnicity, all sites and both sexes combined, 1988–94 patient cohort. Note: based on data from 11 SEER registries that include the states of Connecticut, Hawaii, Iowa, New Mexico, and Utah; and the metropolitan areas of Atlanta, Detroit, Los Angeles, San Francisco and Oakland, San Jose and Monterey, and Seattle.

**Figure 6:**
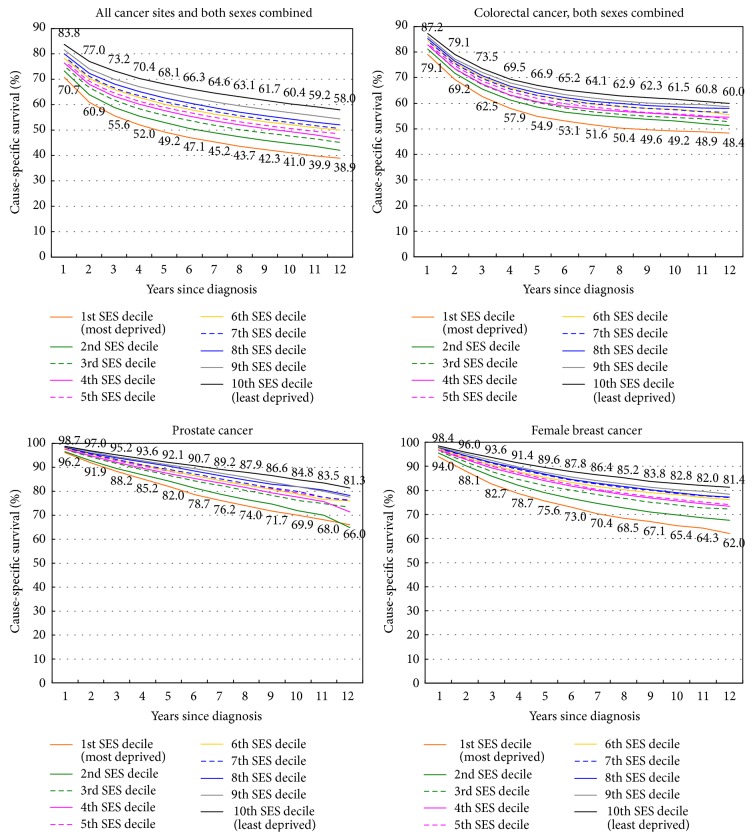
Cancer survival by census tract socioeconomic deprivation index, 11 SEER registries, United States, 1988–1999.

Patient survival rates were significantly lower among men and women in more deprived neighborhoods (Figures 5 and 6). The 5-year survival rate for Black patients diagnosed with cancer was 46% in the most-deprived quintile, significantly lower than the survival rate of 61% for Blacks and 66.0% for non-Hispanic Whites in the least-deprived quintile. Socioeconomic gradients in cancer survival existed for all racial/ethnic groups except AIANs, with Black patients within each SES stratum experiencing lower survival than their non-Hispanic White counterparts (Figure 5). Racial and SES disparities in survival existed even after controlling for stage of disease at diagnosis. Among women diagnosed with localized stage breast cancer during 1988–1994, the 5-year survival rate was 88% for Black women in the most-deprived quintile, compared with the survival rate of 91% for Black women in the least-deprived quintile; this difference in survival rates while statistically significant was relatively small (data not shown).

Differences by deprivation deciles show wide disparities in survival from all-cancers combined and colorectal, prostate, and breast cancer (Figure 6). During 1988–1999, the 10-year survival rate for patients diagnosed with cancer was 41% in the most-deprived decile, compared with 60.4% in the least-deprived decile. The corresponding 10-year survival rates for patients diagnosed with colorectal cancer were 49.2% and 61.5%.

Table 4 shows relative risks of mortality among patients diagnosed with specific cancers during 1988–1999 after adjusting for age and period of diagnosis, sex, race/ethnicity, marital status, area deprivation, and rural-urban residence. Cancer patients in the most-deprived decile had 56% higher adjusted risk of mortality than those in the least-deprived decile. Patient mortality by deprivation levels were particularly pronounced for breast and prostate cancer. After adjusting for deprivation and other covariates, Blacks, American Indians, and Hispanics experienced significantly higher patient mortality than non-Hispanic Whites. Several API groups such as Chinese, Filipinos, Koreans, Vietnamese, and Hawaiians had higher overall patient mortality than non-Hispanic Whites.

### 3.5. Disparities in Risk Factors and Cancer Screening

Marked racial/ethnic and socioeconomic disparities exist in smoking, physical inactivity, dietary behavior, and cancer screening uptake (Table 5) [29]. Compared to non-Hispanic Whites, AIANs and Blacks had higher rates of smoking, obesity, and physical inactivity; APIs had lower rates of smoking and obesity; and Hispanics had higher rates of obesity. AIANs and Hispanics had lower rates of breast, cervical, and colorectal cancer screening compared to Whites. Those with lower education and income levels had significantly higher prevalence of smoking, obesity, physical inactivity, inadequate fruit/vegetable intake, and lower rates of cancer screening.

## 4. Discussion

In this study, we have presented a comprehensive analysis of social inequalities in cancer mortality, incidence, and patient survival from all-cancers combined and from major cancers. New analyses of area- and individual-level socioeconomic disparities in cancer mortality spanning over 6 decades are a particularly novel feature of the study. Analysis of long-term trends and contemporary socioeconomic and racial/ethnic inequalities in cancer adds to the extensive literature on cancer disparities. Socioeconomic and racial patterns in US cancer mortality have reversed over time, and the continued widening of the inverse socioeconomic gradients in all-cancer, lung, and colorectal cancer mortality is consistent with those observed for all-cause and cardiovascular-disease mortality in the US [2, 3, 27].

Consistent with past research, socioeconomic inequalities in cancer incidence and mortality in the US are particularly marked in lung, cervical, stomach, and liver cancer [3–6, 10, 11, 16]. Substantial socioeconomic disparities exist not only in cancer incidence and mortality but also in stage at cancer diagnosis and survival [3, 6, 10, 16, 24]. Such inequalities have been shown to exist for Whites, Blacks, Hispanics, APIs, and AIANs [3, 6, 10]. Incidence and survival analyses pertain to the 1988–1999 period and serve as important benchmarks. However, these analyses need to be updated with more recent data to see if area-based socioeconomic disparities in incidence and survival have persisted or widened over time.

Area-level SES patterns in cancer incidence were generally similar to individual-level patterns derived from the linked SEER-NLMS data [16]. In the linked data, men and women with less than a high school education had 3.0 and 2.0 times higher lung cancer incidence rates, respectively, than those with a college degree [16]. Those below the poverty level had 52–72% higher lung cancer incidence rates than their counterparts with incomes at ≥600% of the poverty level [16]. Individuals with the lowest education and income levels had higher colorectal cancer incidence rates than their most-advantaged counterparts [16]. Higher education and income levels were associated with higher prostate and breast cancer incidence rates [16]. Consistent with the neighborhood level pattern, women with less than a high school education had 3.2 times higher cervical cancer incidence than those with a college degree [16].

Disparities in incidence and mortality from various cancers may reflect differences in smoking prevalence, dietary patterns, obesity, physical inactivity, reproductive factors (e.g., delayed childbearing, childlessness, and breastfeeding), alcohol use, human papillomavirus (HPV) infection, cancer screening, and healthcare factors [3, 5, 6, 12, 15, 20, 30]. Higher smoking rates are more prevalent among men and women in lower SES groups and in more deprived areas (Table 5) [2, 5, 17, 20]. Smoking rates have fallen more rapidly for those in higher SES groups, which largely explains temporal SES trends in all-cancer and lung cancer mortality rates [2, 4–6]. Dietary factors such as fat intake, red meat consumption, and high calorie intake have been mentioned as risk factors for colorectal, prostate, and breast cancer and inequalities in both incidence and mortality may in part reflect differences in these factors [3, 5, 6, 15]. Previous studies as well as data in Table 5 show higher consumption of lower-quality diets and energy-dense foods and lower intakes of fruits and vegetables among lower SES groups but higher total calorie and fat intake among higher SES groups [2, 3, 19].

Healthcare disparities play a prominent role in producing socioeconomic inequalities in mortality from colorectal, prostate, breast, and cervical cancer. Low-SES individuals and residents of more deprived neighborhoods have substantially higher rates of late-stage diagnoses of lung, colorectal, prostate, breast, and cervical cancer and significantly lower rates of cancer survival than their counterparts from more affluent neighborhoods or SES backgrounds [6, 10, 16, 24, 31–37]. Lack of health insurance, limited access to care, and lower rates of regular pap smear, mammography, and colorectal cancer screening among lower SES individuals (as shown in Table 5) and among residents of more disadvantaged areas may account for their higher rates of late-stage cancer diagnoses [2, 3, 6, 33–35]. However, lower cancer survival rates among the disadvantaged may reflect their not only higher rates of late-stage cancer diagnoses, but also less favorable cancer treatment or medical care [3, 6, 35].

Research suggests that SES and area deprivation levels do not fully account for racial/ethnic disparities in cancer incidence, mortality, and outcomes in the US [3, 6, 10, 11, 14]. For example, within each deprivation group, Blacks have higher all-cancer mortality rates than Whites. Indeed, the overall cancer mortality and incidence rates for Blacks in the most-affluent group are similar to or exceed those for Whites in the most-deprived group [3, 6]. Within each SES or deprivation group, Black women have approximately two times higher cervical cancer mortality and 50% higher breast cancer mortality than White women [3, 6]. Black men in each deprivation group have at least two times higher prostate cancer mortality rates than their White counterparts [3, 6]. Such marked racial inequalities may exist partly because Blacks are socially and materially worse off than Whites across different socioeconomic strata [2, 3]. Moreover, they are more likely to be disadvantaged than Whites in health-risk behaviors, healthcare access and use, and cancer treatment and survival within each deprivation group [2, 3, 6].

Detection of cancer at an early, localized stage may be considered a marker for access to healthcare and preventive health services, including cancer screening [6, 26]. Studies have shown significant Black-White and socioeconomic disparities in stage at cancer diagnosis [6, 10]. Within each SES or deprivation group, Blacks have a higher likelihood than Whites of being diagnosed with advanced-stage colorectal, prostate, breast, and cervical cancers [6, 10]. Additionally, even after controlling for stage at diagnosis, Blacks, in each deprivation group, have significantly lower survival rates from colorectal, prostate, breast, and cervical cancer than Whites [6, 10, 38–40].

### 4.1. Comparison with International Patterns

Although studies of cancer inequalities vary widely in their use of socioeconomic measures and coverage of time periods, socioeconomic disparities in US cancer mortality, incidence, and survival reported here are generally consistent with patterns observed for the other industrialized countries [3, 12, 15]. Consistent with the US pattern, all-cancer mortality rates in England during the past decade increased consistently by area deprivation levels [41, 42]. In several European populations, cancer mortality rates were significantly higher among both males and females in lower education groups [12]. Consistent with the US pattern, lung cancer mortality rates in Canada increased in relation to deprivation levels [43]. Higher lung cancer mortality rates were found among men in lower SES groups in many European countries [12, 44]. Inverse socioeconomic gradients in US colorectal cancer mortality rates are compatible with occupational and educational patterns in mortality observed among several European countries [12, 45]. Marked socioeconomic disparities in US cervical cancer mortality reported here are generally consistent with those shown for other industrialized countries. An approximately twofold higher cervical cancer mortality was found among women in low- than high-SES groups in a study that compared inequalities in various low/middle income countries, North America, and Europe, although the magnitude of socioeconomic inequalities was greater in North America than in Europe [43, 46, 47]. Consistent with the US pattern, cancer survival rates have decreased consistently by deprivation levels in the UK, Australia, and New Zealand [36, 48, 49].

### 4.2. Limitations

This study has some limitations. The SES or deprivation indices used in the mortality trend analysis were defined at the county-level, which could vary greatly across census tracts or neighborhoods within a given county. Unfortunately, census tract geocodes are not available in the national mortality database for confidentiality protection of individual information on the death certificate, and the linkage of US mortality records and area-based SES measures is not feasible at the neighborhood level [3–6, 27, 50, 51]. Given the compositional heterogeneity of counties, the association between deprivation and cancer mortality is likely to be underestimated [3–6, 50, 51]. Additionally, the use of 1970 and 1990 census-based deprivation indices to characterize cancer mortality trends from 1950 through 1998 might have influenced SES-specific mortality patterns if socioeconomic position of some counties changed over time. However, previous analyses using county deprivation indices for different censuses/time periods have demonstrated temporal stability of the SES/deprivation index in terms of its relative socioeconomic classification of US counties over time. These analyses have indicated a high degree of correspondence (correlation >0.90) between 1970, 1980, and 1990 SES indices and essentially similar long-term trends in area socioeconomic inequalities in mortality whether trends were based on the 1970, 1980, or 1990 indices for the entire study period [50, 51]. Our analysis indicates correlations among the 1990, 2000, and 2008–2012 deprivation indices to be >0.95. The high correlations among the indices and the relatively stable socioeconomic standing of county deprivation groups indicate that the broad geographical distribution of deprivation and socioeconomic disadvantage in the United States has changed very little over the past several decades [50, 51]. The small degree of area misclassification that may arise from using different SES indices during the study period is unlikely to affect the general trend of changing cancer mortality patterns shown here.

Another limitation concerns conducting a large number of statistical tests without correcting for multiple comparisons. Most of the statistical tests in this study were conducted using the 0.05 level of significance, implying that 5% of the tests could have been significant by chance. Lowering the alpha cutoff to 0.01 may address this problem to a large extent; indeed, the availability of mortality and incidence rates along with their standard errors in Tables 1–3 allows one to compute and evaluate *t*-tests for significance at the 0.01 level. However, lowering the Type 1 error rate by decreasing the alpha level to 0.01 or 0.001 level comes at the cost of increasing Type II error (failing to detect a difference when there really is one).

### 4.3. Conclusions

Cancer is the second leading cause of death in the US and the most prominent cause of death in terms of years of potential life lost [2, 3, 21]. Evidence presented here indicates how cancer disparities contribute greatly to the overall health inequalities in the US. With large socioeconomic and racial/ethnic inequalities in smoking, obesity, and physical inactivity among young people continuing to persist, inequalities in US cancer mortality and incidence are not expected to diminish in the foreseeable future [2, 3]. Efforts to reduce cancer disparities, especially those in lung cancer, therefore might include tobacco control policies at the national and local levels that place greater smoking restrictions or legislate against smoking in public places, ban tobacco marketing, reduce tobacco availability, increase financial and other barriers to smoking, and provide targeted smoking cessation programs for those in socially disadvantaged groups or areas [3, 6]. Healthcare inequalities have also risen in both absolute and relative terms and socioeconomic and racial/ethnic disparities in stage at diagnosis and survival from major cancers have persisted [3, 6]. These trends would also imply continued social inequalities in cancer mortality and incidence in the future. Health policies therefore should enhance access to cancer screening programs among the disadvantaged populations and underserved areas. Lastly, social policy measures aimed at improving the broader social determinants, such as material living conditions and the social and physical environments, are needed to tackle health inequalities in cancer outcomes [3, 6].

## Figures and Tables

**Table 1:** Age-adjusted all-cancer and site-specific cancer mortality rates per 100,000 population and relative risk (RR) of mortality among those aged ≥25 years by race/ethnicity, educational attainment, and poverty status: 1979–1998 National Longitudinal Mortality Study.

	Age-adjusted mortality			Age-adjusted mortality			Age-adjusted mortality		
	Rate	SE	RR	Rate	SE	RR	Rate	SE	RR
	*All cancers combined, male*			*All cancers combined, female*			*Lung cancer, male*		

Race/ethnicity									
Non-Hispanic White	355.67	2.53	1.00	230.37	1.75	1.00	116.79	1.42	1.00
Non-Hispanic Black	471.66	10.06	1.33^*∗*^	274.13	6.05	1.19^*∗*^	146.18	5.50	1.25^*∗*^
American Indian/Alaska Native	334.70	31.37	0.94	255.35	22.74	1.11	119.49	18.55	1.02
Asian/Pacific Islander	229.76	14.00	0.65^*∗*^	144.98	10.42	0.63^*∗*^	55.26	6.80	0.47^*∗*^
Mexican American	261.37	13.35	0.73^*∗*^	150.75	8.60	0.65^*∗*^	65.23	6.81	0.56^*∗*^
Other Hispanic	241.55	13.14	0.68^*∗*^	141.48	8.14	0.61^*∗*^	57.63	6.14	0.49^*∗*^
Educational attainment (years)	(*P*_trend_ for education < 0.01)			(*P*_trend_ for education < 0.01)			(*P*_trend_ for education < 0.01)		
<12	418.15	4.14	1.57^*∗*^	251.18	3.05	1.23^*∗*^	153.05	2.53	2.36^*∗*^
12	351.49	4.44	1.32^*∗*^	228.32	2.59	1.12^*∗*^	111.67	2.38	1.72^*∗*^
13–15	334.57	6.92	1.26^*∗*^	218.20	4.30	1.07	95.40	3.53	1.47^*∗*^
16+	265.88	5.34	1.00	204.40	4.66	1.00	64.94	2.56	1.00^*∗*^
Poverty status^1^	(*P*_trend_ for income < 0.01)			(*P*_trend_ for income < 0.01)			(*P*_trend_ for income < 0.01)		
<100%	425.92	8.01	1.43^*∗*^	264.01	4.60	1.26^*∗*^	151.81	4.82	1.83^*∗*^
100–150%	418.24	7.99	1.40^*∗*^	245.02	5.06	1.17^*∗*^	146.93	4.83	1.77^*∗*^
150–200%	396.53	7.47	1.33^*∗*^	235.06	4.93	1.12^*∗*^	138.87	4.44	1.67^*∗*^
200–400%	360.04	3.99	1.21^*∗*^	225.60	2.74	1.08^*∗*^	115.51	2.20	1.39^*∗*^
400–600%	320.14	5.35	1.07	216.11	3.90	1.03	99.99	2.86	1.20^*∗*^
Above 600%	298.19	6.15	1.00	208.97	4.60	1.00	83.04	3.06	1.00

	*Lung cancer, female*			*Colorectal cancer, male*			*Colorectal cancer, female*		

Race/ethnicity									
Non-Hispanic White	51.68	0.83	1.00	38.77	0.84	1.00	26.67	0.58	1.00
Non-Hispanic Black	56.81	2.76	1.10^*∗*^	41.66	2.99	1.07^*∗*^	34.35	2.14	1.29^*∗*^
American Indian/Alaska Native	44.15	9.48	0.85	29.18	9.64	0.75	31.98	8.13	1.20
Asian/Pacific Islander	26.67	4.55	0.52^*∗*^	29.85	5.05	0.77^*∗*^	13.07	3.26	0.49^*∗*^
Mexican American	19.45	3.15	0.38^*∗*^	24.11	4.00	0.62^*∗*^	16.22	2.88	0.61^*∗*^
Other Hispanic	19.19	3.00	0.37^*∗*^	21.32	3.91	0.55^*∗*^	13.23	2.50	0.50^*∗*^
Educational attainment (years)	(*P*_trend_ for education < 0.01)			(*P*_trend_ for education < 0.01)			(*P*_trend_ for education > 0.05)		
<12	58.82	1.51	1.83^*∗*^	40.81	1.25	1.53^*∗*^	29.21	0.95	1.16
12	52.84	1.22	1.65^*∗*^	40.11	1.52	1.51^*∗*^	25.91	0.89	1.03
13–15	46.06	1.98	1.44^*∗*^	37.27	2.33	1.40^*∗*^	24.45	1.46	0.97
16+	32.08	1.87	1.00	26.65	1.70	1.00	25.12	1.67	1.00
Poverty status^1^	(*P*_trend_ for income < 0.01)			(*P*_trend_ for income < 0.01)			(*P*_trend_ for income < 0.01)		
<100%	58.97	2.24	1.28^*∗*^	39.54	2.41	1.24^*∗*^	29.59	1.44	1.29^*∗*^
100–150%	54.19	2.43	1.17^*∗*^	41.59	2.47	1.31^*∗*^	29.30	1.62	1.28^*∗*^
150–200%	49.28	2.26	1.07	41.12	2.39	1.29^*∗*^	28.20	1.63	1.23
200–400%	48.83	1.27	1.06	39.08	1.33	1.23^*∗*^	25.75	0.93	1.12
400–600%	48.70	1.80	1.05	37.16	1.88	1.17	26.05	1.39	1.13
Above 600%	46.18	2.13	1.00	31.77	2.05	1.00	22.96	1.56	1.00

	*Prostate cancer*			*Breast cancer, female*			*Cervical cancer*		

Race/ethnicity									
Non-Hispanic White	44.43	0.93	1.00	41.72	0.76	1.00	3.91	0.24	1.00
Non-Hispanic Black	97.31	4.77	2.19^*∗*^	46.83	2.51	1.12^*∗*^	8.71	1.08	2.23^*∗*^
American Indian/Alaska Native	43.11	12.00	0.97	35.79	8.14	0.86	6.14	3.09	1.57
Asian/Pacific Islander	22.38	4.81	0.50^*∗*^	17.77	3.51	0.43^*∗*^	0.97	0.69	0.25
Mexican American	38.04	5.47	0.86^*∗*^	25.81	3.28	0.62^*∗*^	4.42	1.42	1.13
Other Hispanic	42.03	6.00	0.95^*∗*^	24.23	3.28	0.58^*∗*^	5.50	1.56	1.41

	*Prostate cancer*			*Breast cancer, female*			*Cervical cancer*		

Educational attainment (years)	(*P*_trend_ for education > 0.05)			(*P*_trend_ for education < 0.01)			(*P*_trend_ for education < 0.05)		
<12	49.50	1.29	1.03	39.76	1.30	0.89	6.76	0.58	2.49^*∗*^
12	43.49	1.74	0.91	41.58	1.11	0.93	3.87	0.34	1.42
13–15	48.18	2.92	1.00	42.24	1.86	0.94	2.76	0.46	1.01
>16	47.97	2.49	1.00	44.88	2.12	1.00	2.72	0.53	1.00
Poverty status^1^	(*P*_trend_ for income > 0.05)			(*P*_trend_ for income < 0.01)			(*P*_trend_ for income < 0.01)		
<100%	45.36	2.51	0.95	44.84	1.98	1.13	8.34	0.90	4.39^*∗*^
100–150%	49.93	2.52	1.04	41.39	2.22	1.04	6.29	0.88	3.31^*∗*^
150–200%	47.62	2.53	0.99	42.70	2.17	1.08	4.99	0.79	2.63^*∗*^
200–400%	49.60	1.58	1.03	41.78	1.19	1.05	3.88	0.36	2.04^*∗*^
400–600%	43.90	2.22	0.92	38.86	1.64	0.98	2.61	0.42	1.37
Above 600%	47.95	2.78	1.00	39.69	1.98	1.00	1.90	0.45	1.00

	*Stomach cancer, male*			*Stomach cancer, female*			*Liver & IBD cancer, male*		

Race/ethnicity									
Non-Hispanic White	9.84	0.42	1.00	4.75	0.24	1.00	6.41	0.33	1.00
Non-Hispanic Black	20.15	2.09	2.05^*∗*^	8.62	1.07	1.81^*∗*^	8.87	1.37	1.38^*∗*^
American Indian/Alaska Native	11.58	5.23	1.18	5.08	2.93	1.07	3.92	2.77	0.61
Asian/Pacific Islander	23.83	4.56	2.42^*∗*^	14.83	3.23	3.12^*∗*^	22.19	4.10	3.46^*∗*^
Mexican American	12.98	2.78	1.32^*∗*^	9.90	2.25	2.08^*∗*^	12.21	2.77	1.90^*∗*^
Other Hispanic	15.18	3.45	1.54^*∗*^	5.87	1.72	1.24^*∗*^	8.88	2.44	1.39^*∗*^
Educational attainment (years)	(*P*_trend_ for education < 0.05)			(*P*_trend_ for education > 0.05)			(*P*_trend_ for education > 0.05)		
<12	14.50	0.76	1.92^*∗*^	7.38	0.51	1.74^*∗*^	8.47	0.60	1.34^*∗*^
12	9.15	0.70	1.21	4.85	0.38	1.14	6.60	0.61	1.05
13–15	8.98	1.12	1.19	3.80	0.57	0.89	7.98	1.04	1.27
16+	7.56	0.92	1.00	4.25	0.68	1.00	6.30	0.76	1.00
Poverty status^1^	(*P*_trend_ for income < 0.01)			(*P*_trend_ for income < 0.05)			(*P*_trend_ for income > 0.05)		
<100%	14.00	1.44	1.65^*∗*^	6.47	0.69	1.20^*∗*^	10.47	1.27	1.43^*∗*^
100–150%	13.89	1.41	1.64^*∗*^	6.64	0.81	1.24^*∗*^	8.43	1.16	1.15
150–200%	11.94	1.30	1.41^*∗*^	6.03	0.79	1.12^*∗*^	7.46	1.05	1.02
200–400%	11.61	0.71	1.37^*∗*^	5.39	0.42	1.00^*∗*^	6.74	0.53	0.92
400–600%	7.69	0.82	0.91	4.18	0.57	0.78	6.29	0.72	0.86
Above 600%	8.46	1.03	1.00	5.37	0.75	1.00	7.34	0.90	1.00

	*Liver & IBD cancer, female*			*Esophageal cancer, male*			*Esophageal cancer, female*		

Race/ethnicity									
Non-Hispanic White	2.93	0.19	1.00	8.65	0.39	1.00	2.24	0.17	1.00
Non-Hispanic Black	4.88	0.81	1.67^*∗*^	17.16	1.87	1.98^*∗*^	5.31	0.83	2.37^*∗*^
American Indian/Alaska Native	6.75	3.95	2.30^*∗*^	5.68	4.13	0.66	2.29	2.29	1.02
Asian/Pacific Islander	4.69	1.82	1.60^*∗*^	5.15	1.95	0.60^*∗*^	0.61	0.61	0.27
Mexican American	4.35	1.50	1.48^*∗*^	6.51	2.08	0.75^*∗*^	0.49	0.49	0.22
Other Hispanic	7.11	1.80	2.43^*∗*^	7.32	2.20	0.85^*∗*^	2.28	1.03	1.02
Educational attainment (years)	(*P*_trend_ for education < 0.01)			(*P*_trend_ for education < 0.01)			(*P*_trend_ for education > 0.05)		
<12	4.20	0.40	1.84^*∗*^	10.22	0.67	1.89^*∗*^	3.02	0.34	1.24
12	3.13	0.30	1.37	10.13	0.71	1.88^*∗*^	2.25	0.26	0.93
13–15	3.08	0.52	1.35	8.01	0.98	1.48^*∗*^	2.76	0.49	1.14
16+	2.28	0.50	1.00	5.40	0.67	1.00	2.43	0.52	1.00
Poverty status^1^	(*P*_trend_ for income < 0.01)			(*P*_trend_ for income < 0.01)			(*P*_trend_ for income > 0.05)		
<100%	4.52	0.59	1.91^*∗*^	13.77	1.48	1.90^*∗*^	3.39	0.55	1.22
100–150%	5.25	0.74	2.22^*∗*^	9.59	1.27	1.32	2.67	0.50	0.96
150–200%	3.44	0.58	1.45	10.72	1.24	1.48	3.32	0.60	1.19
200–400%	2.77	0.31	1.17	8.85	0.61	1.22	2.12	0.26	0.76
400–600%	2.83	0.45	1.19	7.42	0.75	1.02	2.19	0.40	0.78
Above 600%	2.37	0.49	1.00	7.24	0.87	1.00	2.79	0.54	1.00

Mortality rates are age-adjusted to the 2000 US standard population. ^*∗*^*P* < 0.05. ^1^Defined as a ratio of family income to poverty threshold.

**Table 2:** Age-adjusted all-cancer and site-specific cancer mortality rates per 100,000 population and relative risk (RR) of mortality among those aged ≥25 years by race/ethnicity, educational attainment, and poverty status: 2003–2011 National Longitudinal Mortality Study.

	Age-adjusted mortality			Age-adjusted mortality			Age-adjusted mortality		
	Rate	SE	RR	Rate	SE	RR	Rate	SE	RR
	*All cancers combined, male*			*All cancers combined, female*			*Lung cancer, male*		

Race/ethnicity									
Non-Hispanic White	254.50	5.00	1.00	210.50	4.33	1.00	78.50	2.83	1.00
Non-Hispanic Black	350.00	17.00	1.38^*∗*^	209.50	11.50	1.00	114.17	9.83	1.45^*∗*^
American Indian/Alaska Native	406.17	58.50	1.60^*∗*^	284.17	46.67	1.35	151.50	36.33	1.93^*∗*^
Asian/Pacific Islander	149.33	17.67	0.59^*∗*^	159.83	17.33	0.76^*∗*^	36.83	9.00	0.47^*∗*^
Mexican American	143.67	14.00	0.56^*∗*^	127.00	12.67	0.60^*∗*^	27.33	6.33	0.35^*∗*^
Other Hispanic	155.50	16.00	0.61^*∗*^	140.67	13.67	0.67^*∗*^	28.67	7.00	0.37^*∗*^
Educational attainment (years)	(*P*_trend_ for education < 0.01)			(*P*_trend_ for education < 0.01)			(*P*_trend_ for education < 0.01)		
<12	287.50	9.33	1.68^*∗*^	230.33	8.00	1.43^*∗*^	102.83	5.83	2.57^*∗*^
12	288.33	8.33	1.68^*∗*^	202.00	6.17	1.25^*∗*^	89.17	4.67	2.23^*∗*^
13–15	240.17	9.67	1.40^*∗*^	198.33	8.00	1.23^*∗*^	67.33	5.17	1.68^*∗*^
16+	171.33	7.33	1.00	161.33	8.17	1.00	40.00	3.50	1.00^*∗*^
Poverty status^1^	(*P*_trend_ for income < 0.01)			(*P*_trend_ for income < 0.01)			(*P*_trend_ for income < 0.01)		
<100%	332.00	18.33	1.80^*∗*^	232.83	11.50	1.55^*∗*^	119.33	11.17	2.58^*∗*^
100–150%	301.83	15.67	1.64^*∗*^	234.00	11.17	1.55^*∗*^	103.50	9.33	2.23^*∗*^
150–200%	271.33	14.00	1.47^*∗*^	238.83	11.83	1.59^*∗*^	89.67	8.33	1.94^*∗*^
200–400%	260.33	7.67	1.41^*∗*^	209.17	6.83	1.39^*∗*^	78.00	4.33	1.68^*∗*^
400–600%	243.50	10.17	1.32^*∗*^	174.33	9.00	1.16^*∗*^	71.67	5.67	1.55^*∗*^
Above 600%	184.17	8.50	1.00	150.50	8.33	1.00	46.33	4.17	1.00

	*Lung cancer, female*			*Colorectal cancer, male*			*Colorectal cancer, female*		

Race/ethnicity									
Non-Hispanic White	59.83	2.33	1.00	23.50	1.50	1.00	20.00	1.33	1.00
Non-Hispanic Black	52.17	5.83	0.87	43.33	6.17	1.84^*∗*^	24.33	4.00	1.22
American Indian/Alaska Native	54.33	20.50	0.91	9.00	9.00	0.38	40.83	18.17	2.04
Asian/Pacific Islander	36.83	8.33	0.62^*∗*^	6.50	3.67	0.28^*∗*^	19.83	6.33	0.99
Mexican American	21.00	5.17	0.35^*∗*^	18.00	5.00	0.77	14.67	4.33	0.73
Other Hispanic	26.67	6.00	0.45^*∗*^	11.67	4.50	0.50	18.00	5.00	0.90
Educational attainment (years)	(*P*_trend_ for education < 0.01)			(*P*_trend_ for education < 0.01)			(*P*_trend_ for education < 0.01)		
<12	71.33	4.67	2.04^*∗*^	26.67	3.00	1.42^*∗*^	25.67	2.67	2.20^*∗*^
12	60.17	3.33	1.72^*∗*^	26.83	2.67	1.42^*∗*^	19.67	2.00	1.69^*∗*^
13–15	41.33	3.67	1.18^*∗*^	21.50	3.00	1.14	21.50	2.67	1.84^*∗*^
16+	35.00	3.83	1.00^*∗*^	18.83	2.50	1.00	11.67	2.17	1.00
Poverty status^1^	(*P*_trend_ for income < 0.01)			(*P*_trend_ for income > 0.05)			(*P*_trend_ for income < 0.01)		
<100%	67.83	6.33	2.30^*∗*^	20.00	4.67	1.02	22.83	3.67	1.47
100–150%	68.33	6.17	2.32^*∗*^	27.17	4.83	1.38	27.50	3.83	1.77^*∗*^
150–200%	69.33	6.50	2.35	28.33	4.67	1.44	20.83	3.50	1.34
200–400%	57.17	3.50	1.94	25.33	2.50	1.29	23.33	2.33	1.51^*∗*^
400–600%	48.50	4.67	1.64	24.17	3.17	1.23	12.00	2.50	0.77
Above 600%	29.50	3.67	1.00	19.67	2.83	1.00	15.50	2.83	1.00

	*Prostate cancer*			*Breast cancer, female*			*Cervical cancer*		

Race/ethnicity									
Non-Hispanic White	23.50	1.50	1.00	31.67	1.67	1.00	1.67	0.33	1.00
Non-Hispanic Black	54.00	6.83	2.30^*∗*^	35.33	4.83	1.12	2.67	1.33	1.60
American Indian/Alaska Native	41.83	20.83	1.78	38.50	17.17	1.22	13.33	9.50	8.00^*∗*^
Asian/Pacific Islander	6.67	3.83	0.28^*∗*^	11.50	4.67	0.36^*∗*^	1.83	1.83	1.10
Mexican American	8.33	3.83	0.35^*∗*^	16.17	4.50	0.51^*∗*^	2.83	1.67	1.70
Other Hispanic	20.67	6.17	0.88	17.33	4.83	0.55^*∗*^	5.83	2.67	3.50^*∗*^
Educational attainment (years)	(*P*_trend_ for education > 0.05)			(*P*_trend_ for education < 0.05)			(*P*_trend_ for education < 0.01)		
<12	29.33	2.83	1.63^*∗*^	26.17	2.67	0.82	4.17	1.33	6.25^*∗*^
12	23.33	2.50	1.30	29.00	2.33	0.91	3.00	0.83	4.50^*∗*^
13–15	28.00	3.50	1.56^*∗*^	32.83	3.17	1.03	1.33	0.67	2.00
>16	18.00	2.50	1.00	32.00	3.50	1.00	0.67	0.33	1.00
Poverty status^1^	(*P*_trend_ for income < 0.01)			(*P*_trend_ for income > 0.05)			(*P*_trend_ for income < 0.01)		
<100%	31.50	5.67	1.72^*∗*^	29.00	4.17	0.90	4.00	1.67	4.00^*∗*^
100–150%	27.17	4.33	1.48	28.83	4.00	0.90	4.00	1.83	4.00^*∗*^
150–200%	26.50	4.00	1.45	31.00	4.33	0.96	4.83	2.00	4.83^*∗*^
200–400%	24.50	2.33	1.34	31.17	2.67	0.97	2.83	0.83	2.83
400–600%	27.33	3.67	1.49	25.83	3.50	0.80			
Above 600%	18.33	3.17	1.00	32.17	3.83	1.00	1.00	0.50	1.00

^*∗*^ *P* < 0.05. ^1^Defined as a ratio of family income to poverty threshold.

**Table 3:** Age-adjusted incidence rates per 100,000 population for all cancers combined and site-specific cancers by neighborhood (census tract) socioeconomic status (SES) index and race/ethnicity, 1988–1992, 11 SEER registries.

SES index	Male	Male	Male	Male	Male	Male	Female	Female	Female	Female	Female	Female
	All races	All races	White	White	Black	Black	All races	All races	White	White	Black	Black
	Rate	SE	Rate	SE	Rate	SE	Rate	SE	Rate	SE	Rate	SE
*All cancers combined*												
1st quintile	585.07	2.54	555.65	3.18	732.34	5.40	377.44	1.72	384.31	2.26	401.44	3.20
2nd quintile	576.15	2.20	586.13	2.42	681.48	9.40	393.98	1.58	403.35	1.77	392.03	5.48
3rd quintile	595.77	2.19	607.17	2.36	714.31	13.13	409.05	1.53	417.93	1.67	404.77	7.44
4th quintile	594.28	2.21	602.34	2.32	659.73	16.70	417.06	1.54	424.35	1.63	418.02	9.95
5th quintile	588.38	2.22	595.95	2.33	650.70	20.04	431.28	1.58	438.21	1.67	414.62	12.58
RR (Q1/Q5)	0.99		0.93^*∗*^		1.13^*∗*^		0.88^*∗*^		0.88^*∗*^		0.97	
*P*_trend_	>0.05		0.05		<0.01		<0.01		<0.01		<0.05	

*Lung cancer*												
1st quintile	115.31	1.11	105.40	1.38	156.51	2.43	47.86	0.61	47.73	0.80	55.35	1.18
2nd quintile	101.20	0.92	101.05	1.00	137.38	4.13	45.19	0.53	46.58	0.60	48.98	1.92
3rd quintile	98.81	0.88	101.15	0.95	118.06	5.07	48.69	0.53	50.75	0.58	47.39	2.56
4th quintile	92.25	0.86	93.65	0.91	105.23	6.21	49.12	0.53	50.61	0.56	49.05	3.42
5th quintile	71.80	0.77	72.53	0.81	85.07	6.89	45.35	0.51	46.93	0.54	43.85	4.30
RR (Q1/Q5)	1.61^*∗*^		1.45^*∗*^		1.84^*∗*^		1.06^*∗*^		1.02		1.26^*∗*^	
*P*_trend_	<0.01		<0.01		<0.01		>0.05		>0.05		<0.01	

*Colorectal cancer*												
1st quintile	66.03	0.87	63.73	1.10	77.24	1.79	47.46	0.60	44.60	0.75	56.35	1.20
2nd quintile	72.64	0.79	74.28	0.87	72.75	3.10	50.68	0.55	50.69	0.60	59.11	2.20
3rd quintile	73.00	0.78	72.72	0.83	89.14	4.73	50.15	0.53	50.13	0.56	59.76	3.00
4th quintile	73.41	0.80	73.86	0.83	75.50	5.90	49.76	0.53	49.62	0.55	63.53	4.07
5th quintile	70.84	0.80	71.03	0.83	64.51	6.37	49.35	0.54	49.03	0.57	60.84	5.05
RR (Q1/Q5)	0.93^*∗*^		0.90^*∗*^		1.20		0.96^*∗*^		0.91^*∗*^		0.93	
*P*_trend_	>0.05		>0.05		>0.05		>0.05		>0.05		<0.01	

*Stomach cancer*												
1st quintile	20.98	0.49	18.23	0.58	24.91	1.01	9.73	0.27	8.57	0.33	10.37	0.52
2nd quintile	13.95	0.35	12.33	0.36	21.03	1.70	6.45	0.20	5.46	0.20	9.68	0.90
3rd quintile	14.35	0.35	13.01	0.35	21.68	2.36	6.28	0.19	5.48	0.18	10.74	1.34
4th quintile	13.60	0.35	12.68	0.35	16.25	2.76	6.10	0.18	5.48	0.18	10.99	1.71
5th quintile	12.94	0.34	11.64	0.34	20.27	3.65	5.75	0.19	4.98	0.18	9.64	2.11
RR (Q1/Q5)	1.62^*∗*^		1.57^*∗*^		1.23		1.69^*∗*^		1.72^*∗*^		1.08	
*P*_trend_	<0.05		<0.05		>0.05		<0.05		<0.05		>0.05	

*Liver and intrahepatic bile duct cancer*												
1st quintile	10.39	0.33	7.78	0.38	9.52	0.59	3.37	0.16	2.66	0.19	3.33	0.29
2nd quintile	6.35	0.23	4.96	0.22	8.98	1.03	2.45	0.12	2.16	0.13	2.98	0.51
3rd quintile	5.73	0.21	4.78	0.21	7.22	1.24	2.30	0.11	1.83	0.11	2.45	0.58
4th quintile	5.07	0.20	4.43	0.20	6.21	1.39	1.96	0.10	1.74	0.10	3.94	1.02
5th quintile	5.02	0.20	3.98	0.19	8.18	2.37	2.04	0.11	1.75	0.11	0.84	0.63
RR (Q1/Q5)	2.07^*∗*^		1.95^*∗*^		1.16		1.65^*∗*^		1.52^*∗*^		3.96	
*P*_trend_	<0.01		<0.01		>0.05		<0.01		<0.01		>0.05	

*Esophageal cancer*												
1st quintile	11.54	0.35	7.51	0.37	22.04	0.90	3.47	0.17	2.21	0.17	6.44	0.41
2nd quintile	7.17	0.25	6.72	0.26	13.25	1.25	1.82	0.11	1.71	0.11	3.17	0.48
3rd quintile	6.94	0.23	6.68	0.25	11.99	1.61	2.04	0.11	2.05	0.11	3.71	0.74
4th quintile	6.68	0.23	6.72	0.24	8.93	1.70	2.01	0.11	2.03	0.11	2.50	0.71
5th quintile	5.49	0.21	5.39	0.22	8.50	2.20	2.13	0.11	2.21	0.12	4.06	1.41
RR (Q1/Q5)	2.10^*∗*^		1.39^*∗*^		2.59^*∗*^		1.63^*∗*^		1.00		1.59	
*P*_trend_	<0.01		<0.01		<0.01		>0.05		>0.05		>0.05	

*Breast cancer (female)*							*Cervical cancer*					
1st quintile	101.38	0.90	103.29	1.20	110.96	1.70	19.71	0.40	21.47	0.55	18.51	0.68
2nd quintile	119.20	0.89	122.64	1.00	116.61	2.92	12.13	0.29	12.07	0.32	13.34	0.98
3rd quintile	127.43	0.87	130.31	0.95	123.28	3.95	10.50	0.25	9.91	0.27	14.62	1.31
4th quintile	134.09	0.88	137.23	0.94	124.95	5.20	8.55	0.22	8.31	0.23	11.53	1.47
5th quintile	148.53	0.92	152.04	0.98	134.45	6.69	7.41	0.20	7.00	0.21	14.22	2.09
RR (Q1/Q5)	0.68^*∗*^		0.68^*∗*^		0.83^*∗*^		2.66^*∗*^		3.07^*∗*^		1.30	
*P*_trend_	<0.01		<0.01		<0.01		<0.01		<0.01		>0.05	

*Prostate cancer*												
1st quintile	154.11	1.34	133.67	1.61	229.51	3.12						
2nd quintile	164.11	1.19	164.57	1.29	240.49	5.86						
3rd quintile	178.61	1.22	181.46	1.31	261.93	8.41						
4th quintile	179.84	1.24	181.05	1.29	254.85	10.87						
5th quintile	195.63	1.32	197.32	1.37	280.15	13.76						
RR (Q1/Q5)	0.79^*∗*^		0.68^*∗*^		0.82^*∗*^							
*P*_trend_	<0.01		<0.01		<0.01							

Incidence rates are age-adjusted to the 2000 US standard population. RR = rate ratio (SES quintile 1/SES quintile 5). ^*∗*^*P* < 0.05.

Quintile 1 represents low SES and high deprivation level, whereas quintile 5 denotes high SES and low deprivation level.

The 11 SEER registries include the states of Connecticut, Hawaii, Iowa, New Mexico, and Utah; and the metropolitan areas of Atlanta, Detroit, Los Angeles, San Francisco and Oakland, San Jose and Monterey, and Seattle.

**Table 4:** Multivariate Cox regression models showing adjusted^1^ relative risks (hazard ratios) of mortality among cancer patients diagnosed during 1988–1999, 11 SEER registries (maximum mortality follow-up of 11 years).

	All cancer sites and both sexes combined^2^			Colorectal cancer both sexes combined^3^			Prostate cancer^4^			Female breast cancer^5^		
	Hazard ratio	95% confidence interval		Hazard ratio	95% confidence interval		Hazard ratio	95% confidence interval		Hazard ratio	95% confidence interval	
*Race/ethnicity*												
Non-Hispanic White	1.00	Reference		1.00	Reference		1.00	Reference		1.00	Reference	
Non-Hispanic Black	1.20	1.19	1.21	1.18	1.14	1.23	1.43	1.37	1.50	1.60	1.53	1.67
Hispanic/Latino	1.05	1.03	1.06	1.09	1.04	1.13	1.16	1.09	1.24	1.16	1.10	1.23
American Indian	1.46	1.40	1.54	1.40	1.18	1.67	1.85	1.48	2.31	1.52	1.22	1.90
Chinese	1.22	1.20	1.25	0.92	0.86	0.99	0.86	0.74	1.00	0.91	0.80	1.04
Japanese	0.97	0.94	0.99	0.80	0.75	0.85	0.72	0.64	0.82	0.60	0.53	0.69
Filipino	1.04	1.01	1.06	0.97	0.88	1.05	1.01	0.90	1.13	1.03	0.92	1.15
Hawaiian	1.40	1.34	1.46	1.17	1.01	1.37	1.67	1.31	2.11	1.16	0.97	1.39
Korean	1.54	1.48	1.60	0.91	0.78	1.07	1.19	0.82	1.74	1.00	0.75	1.32
Asian Indian	0.90	0.83	0.98	0.58	0.39	0.86	1.47	1.03	2.09	1.17	0.85	1.61
Vietnamese	1.47	1.41	1.54	0.80	0.64	1.00	1.71	1.14	2.55	1.11	0.83	1.50
Other API	1.14	1.09	1.18	0.81	0.68	0.97	1.15	0.89	1.49	1.12	0.91	1.38

*Neighborhood (census tract) SES index*												
1st decile (low SES)	1.56	1.54	1.59	1.29	1.23	1.36	1.57	1.46	1.68	1.68	1.57	1.79
2nd decile	1.47	1.46	1.49	1.21	1.16	1.27	1.53	1.43	1.63	1.59	1.49	1.69
3rd decile	1.39	1.37	1.41	1.19	1.14	1.24	1.38	1.30	1.46	1.49	1.41	1.58
4th decile	1.34	1.33	1.36	1.17	1.12	1.22	1.28	1.21	1.36	1.40	1.32	1.48
5th decile	1.31	1.29	1.32	1.15	1.11	1.20	1.26	1.19	1.33	1.35	1.28	1.42
6th decile	1.26	1.25	1.28	1.10	1.06	1.15	1.26	1.19	1.34	1.27	1.20	1.34
7th decile	1.23	1.21	1.24	1.10	1.06	1.15	1.25	1.18	1.33	1.21	1.14	1.27
8th decile	1.20	1.18	1.21	1.08	1.04	1.13	1.18	1.11	1.26	1.19	1.13	1.26
9th decile	1.12	1.11	1.13	1.05	1.01	1.10	1.11	1.04	1.18	1.14	1.07	1.20
10th decile (high SES)	1.00	Reference		1.00	Reference		1.00	Reference		1.00	Reference	

^1^Adjusted for age at diagnosis, period of diagnosis, sex, race/ethnicity, marital status, area SES, and rural-urban residence.

^2^Number of diagnosed cancer patients = 1,663,844; number dying during 1988–1999 follow-up = 541,427.

^3^Number of diagnosed colorectal cancer patients = 150,330; number dying during 1988–1999 follow-up = 46,673.

^4^Number of diagnosed prostate cancer patients = 228,839; number dying during 1988–1999 follow-up = 422,784.

^5^Number of diagnosed female breast cancer patients = 197,270; number dying during 1988–1999 follow-up = 24,976.

**Table 5:** Prevalence (%) of current smoking, obesity, physical inactivity, fruit/vegetable intake, and cancer screening by race/ethnicity, education, and income/poverty level in the United States: The 2014-2015 National Health Interview Survey.

	Current smoking male	Current smoking female	Obesity male	Obesity female	Physical inactivity male	Physical inactivity female	Fruit/vegetable intake <1 time per day male	Fruit/vegetable intake <1 time per day female	Mammogram within the past 2 years^1^	Pap test within the past 3 years^1^	Ever had a Colonoscopy^1^
*Race/ethnicity*											
All races	17.9	14.4	31.6	30.8	29.5	32.6	33.7	25.3	73.2	82.7	65.3
Non-Hispanic White	18.2	16.6	31.9	28.6	27.4	29.8	33.4	24.9	72.4	81.5	69.2
Non-Hispanic Black	23.0	14.4	36.5	47.7	33.4	42.1	39.8	31.0	77.8	86.1	61.3
American Indian/Alaska Native	27.4	29.7	45.7	51.1	39.5	35.4	35.5	32.4	68.5	74.0	51.3
Asian/Pacific Islander	14.1	3.8	11.2	12.2	23.5	28.2	23.9	15.1	70.8	85.3	50.8
Hispanic	14.0	7.8	33.8	34.1	38.0	39.4	34.5	26.4	74.9	84.3	48.8

*Educational attainment (years)*											
<High school	27.2	20.1	31.7	37.1	51.1	53.4	35.4	27.9	63.1	74.1	50.6
High school graduate	25.7	20.1	36.0	34.8	38.0	43.3	37.9	29.3	68.7	76.2	62.6
Some college/associate degree	19.7	17.0	37.0	35.2	28.0	31.2	35.4	26.6	73.7	82.4	66.4
≥College graduate	7.2	5.7	23.8	21.8	15.7	17.8	29.7	20.5	80.2	89.0	73.4

*Poverty status (ratio of family income to poverty threshold)*											
<100%	34.1	26.4	31.7	40.4	46.2	47.8	38.5	29.3	64.1	76.8	48.0
100–200%	26.2	19.5	31.5	38.2	41.0	44.1	37.3	28.6	64.3	77.9	55.4
200–300%	19.8	15.9	33.9	33.1	34.9	36.2	33.4	25.9	69.1	80.5	64.4
300–400%	18.9	12.3	33.8	32.3	27.6	28.9	36.9	25.7	74.3	83.3	69.8
400–500%	13.3	9.4	31.7	26.7	24.7	25.4	34.6	24.5	78.0	87.4	70.7
Above 500%	9.7	7.8	30.0	21.6	16.1	16.9	29.5	21.0	81.0	88.2	73.7

Note: ages were ≥40 years for mammography, 25–64 years for Pap test, ≥50 years for colonoscopy, and ≥25 years for smoking, obesity, physical inactivity, and fruit/vegetable intake. Differences in prevalence for each risk factor or screening variable across race/ethnicity, education, and poverty level were statistically significant at *P* < 0.05.

^1^2015 National Health Interview Survey, Cancer Control Supplement.
